# Chemotherapy-induced peripheral neuropathy and its determinants among adult cancer patients on chemotherapy in northwest Ethiopia oncology centers, 2022

**DOI:** 10.3389/fonc.2024.1420518

**Published:** 2024-12-10

**Authors:** Tesfaye Engdaw Habtie, Melsew Dagne Abate, Gebremeskel Kibret Abebe, Zenaw Tessema Wolie, Addis Wondmagegn Alamaw, Haymanot Zeleke Mitiku

**Affiliations:** ^1^ Department of Nursing, College of Health Sciences, Woldia University, Woldia, Ethiopia; ^2^ Department of Adult Health Nursing, College of Health Sciences, Injibara University, Injibara, Ethiopia; ^3^ Department of Emergency and Critical Care Nursing, School of Nursing, College of Health Sciences, Woldia University, Woldia, Ethiopia; ^4^ Department of Pharmacology, College of Health Sciences, Debre Markos University, Debre Markos, Ethiopia; ^5^ Nursing Department, College of Health Sciences, Debre Markos University, Debre Markos, Ethiopia

**Keywords:** cancer, chemotherapy, Ethiopia, peripheral neuropathy, risk factors

## Abstract

**Introduction:**

Peripheral neuropathy is a nerve disorder that causes pain, numbness, and tingling in different parts of the body. It is a major and common clinical problem associated with several chemotherapeutic medications frequently used in cancer treatment, with prevalence rates ranging from 19% to 85%. To the best of the authors’ knowledge, there is a lack of data on the magnitude and determinants of chemotherapy-induced peripheral neuropathy in Ethiopia.

**Objective:**

This study aimed to assess the magnitude and associated factors of chemotherapy-induced peripheral neuropathy among adult cancer patients undergoing chemotherapy in northwest Ethiopia oncology centers in 2022.

**Method:**

An institutional-based cross-sectional study was conducted on 406 eligible adult cancer patients undergoing chemotherapy in northwest Ethiopia oncology centers, between May and July 2022.

**Results:**

Out of 406 adult cancer patients included in the study, 54.4% had peripheral neuropathy. The stage of cancer (AOR = 4.36 [95% CI: 1.76; 10.8]), comorbidity (AOR = 2.74 [95% CI: 1.28; 5.83]), drug regimen (AOR = 2.99 [95% CI: 1.36; 6.54]), and cycle of therapy (AOR = 4.00 [CI: 1.67; 9.65]) were significantly associated with the magnitude of chemotherapy-induced peripheral neuropathy.

**Conclusion:**

Chemotherapy-induced peripheral neuropathy is a common adverse event among Ethiopian cancer patients treated with various chemotherapeutic drugs. Therefore, it is necessary to establish more effective diagnostic methods and incorporate validated assessment tools, such as the European Organization for Research and Treatment of Cancer tools, either alone or in combination with other clinical instruments, into the routine evaluation of all patients receiving chemotherapeutic drug.

## Introduction

Chemotherapy-induced peripheral neuropathy (CIPN) is a major clinical problem among cancer patients undergoing chemotherapy, leading to symptoms such as pain, numbness, tingling, and weakness in the hands and feet (1). It results from damage to the peripheral nerves, which are responsible for transmitting signals between the periphery and the brain (2, 3). CIPN can also contribute to a decreased quality of life due to complications such as foot ulcers, pain, and muscle weakness (4). CIPN can be acute, lasting for a few months, or chronic, lasting more than 6 months (1, 5).

Chemotherapy-induced peripheral neuropathy is a major problem for cancer patients, their care providers, and the healthcare system worldwide (6, 7). Its prevalence varies depending on the type of chemotherapy drugs used (8–11), but it is estimated to affect up to 48% of cancer patients annually (5, 9). In the USA, approximately 68.4% of cancer survivors develop CIPN (12), while in Africa, including Ethiopia, the prevalence of CIPN is not well studied. However, the burden of the disease has increased in recent years, placing significant strain on the healthcare systems due to premature death, high costs, and complications arising from therapies (13, 14).

The average healthcare costs for cancer patients undergoing chemotherapy with PN increased by $8,092 compared to those without PN. On average, each case of CIPN resulted in 12 additional outpatient visits and more days spent in the hospital (15). CIPN is not only a theft of money and time; rather, it is also a theft of equipment and human resources, including medical oncologists, primary care physicians, physician assistants, nurses, neurologists, and pain specialists, who can participate at the same time or in later steps (16). Despite the increased burden of CIPN, there is limited attention given to its assessment, leading to significant underreporting of its prevalence (6, 7). To the best of the authors’ knowledge, there is a lack of data on the magnitude and determinants of chemotherapy-induced peripheral neuropathy in Ethiopia (17). Consequently, the extent of CIPN remains poorly understood in developing countries, particularly in sub-Saharan Africa, including Ethiopia. The study conducted in East Africa, specifically Kenya, highlights the lack of data regarding the magnitude of CIPN among cancer patients undergoing chemotherapy (17). CIPN may manifest differently in Ethiopia compared to other regions due to several interrelated factors. Genetic diversity, including variations in the CYP450 enzyme in African populations, can influence drug metabolism, potentially altering the risk and severity of CIPN. Additionally, unmeasured levels of nerve-protective nutrients such as vitamins B and E and magnesium in Ethiopia may increase vulnerability to neuropathy. Limited access to healthcare, cost-driven chemotherapeutic regimens (e.g., platinum-based drugs or taxanes), and a lack of supportive therapies can delay the detection and management of CIPN, thereby increasing the risk and severity of its symptoms. Assessing the magnitude and associated factors of CIPN is crucial for early identification, which can help mitigate related health, socioeconomic, and healthcare expenditure issues.

## Method

### Study design and area

An institutional-based cross-sectional study was conducted between 30 May 30 and 30 July 2022 in northwest Ethiopia oncology centers, which is located in the northwestern part of Ethiopia. Of the 96 government-affiliated and private hospitals in the region, only four had oncology centers with a total of 68 beds during the study period. The total number of cancer patients undergoing chemotherapy during the study period were 920 (18).

### Eligibility criteria

All adult cancer patients undergoing chemotherapy during the data collection period were included in the study, while patients with neurological diseases (stroke, Parkinson’s disease, or Alzheimer’s disease), HIV patients on Highly Active Antiretroviral Therapy (HAART), TB patients on anti-TB, diabetic patients (19), and those receiving their first cycle of chemotherapy (20) where excluded from the study.

### Sampling

The sample size was calculated using the single population proportion formula, with the following assumptions to obtain the maximum sample size: *p* = 50% (due to the absence of prior studies on this topic in Ethiopia at the time of this thesis), a confidence level of 95%, and margin of error (*d*) of 5%. After including a 10% allowance for nonresponses, the final sample size was determined to be 423. The sample size was then proportionally allocated based on the population size of each public hospital with oncology centers. Before data collection began, cancer patients undergoing chemotherapy who had neurological diseases, HIV patients on HAART, TB patients on anti-TB, and diabetic patients were excluded from the database. Subsequently, the sampling frame was prepared based on the appointment list for the study period. Finally, the study subjects were selected using a simple random sampling technique, employing a lottery method.

### Operational definitions

- CIPN: is defined as peripheral neuropathy that develops after chemotherapy. For patients with pre-existing peripheral neuropathy, PN is considered chemotherapy-induced only if the symptoms worsen, as reported by the patient according to the European Organization for Research and Treatment of Cancer (EORTC) QLQ-PN20 response categories, following the initiation of chemotherapy (21).
- The magnitude of PN is defined as the percentage of patients who reported at least one neuropathy symptom that bothered them, based on the EORTC QLQ-PN20 response categories. Respondents who scored at least one were classified as having peripheral neuropathy, while those who scored below one were classified as not having PN (22).
- We employed the EORTC QLQ-CIPN20 tool, which specifically measures the magnitude of chemotherapy-induced peripheral neuropathy through a series of validated questions related to sensory, motor, and autonomic symptoms. While we collected subjective reports from patients regarding their symptoms, we also utilized the Common Terminology Criteria for Adverse Events (CTCAE) to ensure a standardized assessment of severity. The tool provides a comprehensive overview of the patient’s experience with CIPN, allowing us to quantify the impact of neurotoxicity on their quality of life.
- Current smoker: an adult who has currently smoked cigarettes sometimes or more than once a week (23).
- Former smoker: an adult who has smoked at least 100 cigarettes in his or her lifetime but who had currently quit smoking (24).

According to IPAQ-SF tool, physical activity is classified as low, moderate, or high.

- High is defined as meeting one of the following two criteria: performing vigorous-intensity activities, such as heavy lifting, aerobics, digging, and plowing, on at least 3 days a week.
- Moderate is defined as meeting one of the following three criteria: engaging 3 or more days of vigorous activity for at least 20 min/day; 5 or more days of moderate-intensity activity or walking for at least 30 min/day; or 5 or more days of any combination of walking.
- Low refers to individuals who do not meet the criteria for either the high or moderate categories and are considered inactive (25).
- Alcohol user: for men, a score of 4 or more is considered positive for alcohol use, and for women, a score of 3 or more is considered positive, according to the AUDIT response (26).

### Data collection procedures and tools

Data were collected from the patients’ charts and through an adapted, pretested, structured, interviewer-administered questionnaire. The questionnaire included sociodemographic characteristics, behavioral traits, clinical parameters, and a brief section adapted from the EORTC quality of life tool. It was administered by four nurses with bachelor’s degrees. The questionnaire is a multi-item scale not limited to any specific disease or treatment group. The current version of CIPN20 includes three scales to assess the symptoms and function: sensation (nine items), motor (eight items), and autonomic nerves (three items) (27, 28). The questionnaire was first developed in English and then translated into Amharic by an expert. It was subsequently translated back into English by an independent translator to ensure consistency, and was used to collect the necessary data for CIPN assessment. To ensure the quality of the data, a pretest was conducted with 5% of the total sample size, consisting of cancer patients receiving chemotherapy at Black Lion Hospital (which was not a study area), and those participants were excluded from the actual sample. Necessary adjustments were made to the tool based on the pretest results. The reliability of the tool was checked before data collection, yielding alpha coefficients of 0.93 for the sensory scale, 0.85 for the motor scale, and 0.78 autonomic scale. Each questionnaire and data sheet was checked before data entry. Data were entered daily for nearby sites and weekly for more distant sites, with no major missing data identified.

### Statistical analysis

Data were entered into EPI-Data Version 4.6 and then transferred to SPSS version 25.0 for analysis. Bivariable binary logistic regression was performed to identify the independent factors associated with CIPN. The adequacy of the model was tested using Hosmer and Lemeshow’s goodness-of-fit test, with a *p*-value of 0.209.

Variables with a *p*-value < 0.25 in the bivariable analysis were entered into a multivariable binary logistic regression model using the backward LR method. A *p*-value < 0.05 and odds ratio (OR) with a 95% confidence interval (CE) was considered statistically significant in this study. The results were presented using tables, graphs, and narrative descriptions.

## Result

### Sociodemographic characteristics

A total of 423 adult cancer patients receiving chemotherapy were selected to participate in this study, and 406 adults participated, resulting in a response rate of 96%.

About 252 (62.1%) of respondents were females. Regarding age 145(35.7%) of the participants were within the age range of 35 to 49 years with a minimum of 22 and a maximum of 75 years. The mean age of the participant was 48.56 ± 13.6 years. In terms of educational status about, 158 (38.9%) of the respondents were unable to read and write followed by 107 (26.4%) of respondents learnt secondary school. Only 79 (19.5%) of the respondents learn higher education. According to their place of residence, about 225 respondents (55.4 percent) were urban dwellers (**Table 1**).

**Table 1:** Sociodemographic and related clinical characteristics of adult cancer patients receiving chemotherapy in northwest Ethiopia oncology centers, 2022 (*N* = 406).

Sociodemographic and related variables	Frequency (*N*)	Percent
Gender		
Men	154	37.9%
Women	252	62.1%
Age		
18–34	67	16.5%
35–49	145	35.7%
50–64	128	31.5%
≥ 65	70	17.2%
Resident of the participant		
Urban	225	55.4%
Rural	181	44.6%
Educational status		
Cannot write and read	158	38.9%
Primary	62	15.3%
Secondary	107	26.4%
Higher education	79	19.5%

### Behavioral characteristics of the respondents

Regarding behavioral characteristics, approximately 390 respondents (96.1%) did not smoke cigarettes, 231 respondents (56.9%) engaged in moderate physical exercise, and 361 respondents (88.9%) were nonalcoholic (**Table 2**).

**Table 2:** Behavioral and clinical characteristics of adult cancer patients receiving chemotherapy in northwest Ethiopia oncology centers, 2022 (*N* = 406).

Variable	Category	Frequency (*N*)	Percent
Smoking status of the respondent	Nonsmoker	390	96.1%
	Previous smoker	12	3.0%
	Current smoker	4	1.0%
Physical activity of the respondent	Low	28	6.9%
	Moderate	231	56.9%
	High	147	36.2%
Alcoholic status of the respondent	No	361	88.9%
	Yes	45	11.1%

### Clinical and related characteristics of the respondents

**Figure 1:**
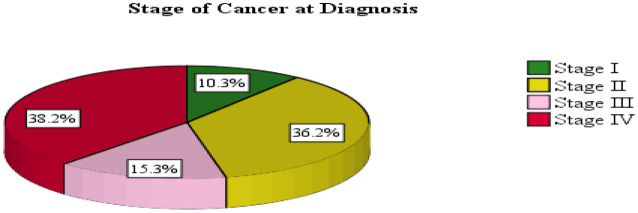
Cancer stage at chemotherapy initiation among adult patients in northwest Ethiopia oncology centers, 2022.

The primary diagnoses in this study group were breast tumors (*n* = 128; 31.5%), cervical tumors (*n* = 53; 13.1%), lung tumors (*n* = 29; 7.1%), non-Hodgkin lymphomas (*n* = 23; 5.7%), and colon cancers (*n* = 21; 5.2%). At the time of chemotherapy initiation, 155 patients (38.2%) were in stage 4, while 147 patients (36.2%) were in stage 2 (**Table 3**; **Figure 1**).

**Table 3:** Types and magnitude of cancer among patients in northwest Ethiopia oncology centers, 2022 (*N* = 406).

Type of cancer	Frequency (*N*)	Percent
Breast cancer	128	31.5%
Cervical cancer	53	13.1%
Lung cancer	29	7.1%
Non-Hodgkin lymphoma	23	5.7%
Colonic cancer	21	5.2%
Ovarian cancer	18	4.4%
Soft tissue sarcoma	17	4.2%
Esophageal cancer	14	3.4%
Colorectal cancer	13	3.2%
Osteosarcoma	12	3%
Lymphocytic leukemia	11	2.7%
Gastric cancer	9	2.2%
Pancreatic cancer	9	2.2%
Prostate cancer	7	1.7%
Cholangiocarcinoma	7	1.7%
Hepatocellular carcinoma	6	1.5%
Nasopharyngeal cancer	6	1.5%
Thyroid cancer	6	1.5%
Gestational trophoblastic neoplasia	6	1.5%
Hodgkin lymphoma	6	1.5%
Skin cancer	5	1.2%

Regarding comorbidity, of the 406 respondents, 61 (15%) had noncommunicable chronic diseases, with hypertension accounting for approximately 46 cases (75.4%). Additionally, 115 respondents (28.3%) were classified as underweight.

Concerning the therapies used to treat the neoplasms included in the study, the taxane regimen (*n* = 113; 27.8%), consisting of paclitaxel (*n* = 58; 14.3%) and docetaxel (*n* = 55; 13.5%), was the most commonly prescribed chemotherapeutic drug. This was followed by alkylating agents with anthracyclines (cyclophosphamide combined with doxorubicine *n* = 96; 23.6%) and platinum-based regimens, either alone or in combination (*n* = 80; 19.7%). Among the respondents, 117 (28.8%) were on the second cycle of therapy (**Table 4**).

**Table 4:** Frequency of comorbidities, chemotherapeutic drugs, and therapy cycles among adult cancer patients receiving chemotherapy in northwest Ethiopia oncology centers, 2022 (*N* = 406).

Name of variables	Categories	Frequency (*N*)	Percent
BMI	Underweight	115	28.3%
	Normal weight	251	61.8%
	Over weight	40	9.9%
Drug regimen	Alkylating agent with anthracycline	96	23.6%
	Taxane regimen alone	113	27.8%
	Paclitaxel	58	14.3%
	Docetaxel	55	13.5%
	Taxane regimen with combination	57	14%
	Platinum alone and with combination	80	19.7%
	Vinca alkaloids	42	10.3%
	Others	18	4.4%
Cycle of therapy	Second cycle	117	28.8%
	Third cycle	98	24.1%
	Fourth cycle	49	12.1%
	Fifth cycle	71	17.5%
	Sixth and above	71	17.5%
Comorbidity	Yes	61	15%
	No	345	85%

### Magnitude of chemotherapy-induced peripheral neuropathy

Among the respondents analyzed, 221 (54.4%) had chemotherapy-induced peripheral neuropathy, with a 95% CI of 49.6% to 59.3%. The proportion of sensory PN was higher, affecting 218 (53.7%) of the participants, followed by motor symptoms in 186 (45.8%). Among the sensory manifestations, the most frequently reported symptoms were tingling, burning sensations, and numbness, particularly in the hands and, especially, the legs. Walking difficulties were the most prevalent motor symptoms.

### Factors associated with the magnitude of chemotherapy-induced peripheral neuropathy

#### Bivariable and multivariable analysis of CIPN and associated factors

To determine factors associated with the magnitude of CIPN (dependent variable), bivariable logistic regression was performed. Significant variables, including sex, stage of cancer at diagnosis, comorbidity, physical activity, age, body mass index, type of chemotherapeutic drugs, and cycle of therapy, were found to be associated with PN. Variables with a *p*-value of < 0.25 were included in the multivariable logistic regression model to adjust for possible confounders.

Multivariable logistic regression was then performed using a backward stepwise (likelihood ratio) method to ascertain the effect of the independent variables on CIPN. The results showed that the stage of cancer at diagnosis, comorbidity, type of chemotherapeutic drug, and cycle of therapy were significantly associated with PN (**Table 5**).

**Table 5:** Factors associated with the magnitude of CIPN among adult cancer patients receiving chemotherapy in northwest Ethiopia oncology centers, 2022.

Variable	Category	Frequency (*N*) of PN		COR (95% CI)	AOR (95%)	*p*-value
		No	Yes			
Gender	Men	85	69	1	1	
	Women	100	152	1.87 (1.25; 2.81)^**^	1.58 (.93; 2.6)	0.09
Stage of cancer at diagnosis	I	30	12	1	1	
	II	90	57	1.58 (.75; 3.34)	1.27 (.54; 2.94)	0.59
	III	34	28	2.06 (.89; 4.75)	1.55 (.60; 3.96)	0.37
	IV	31	124	10.0 (4.60; 21.74)^**^	4.36 (1.76; 10.80)^**^	**0.001**
Comorbidity	No	172	173	1	1	
	Yes	13	48	3.67 (1.92; 7.02)^**^	2.74 (1.28; 5.83)^**^	**0.009**
Physical activity	Low	16	12	1	1	
	Moderate	127	104	1.09 (0.50; 2.41)	1.05 (0.39; 2.81)	0.93
	High	42	105	3.33 (1.45; 7.64)^**^	2.42 (0.87; 6.74)	0.091
Age	18–34	32	31	1	1	
	35–49	67	78	1.20 (0.67; 2.17)	0.81 (0.39; 1.70)	0.586
	50–64	63	65	1.07 (0.58; 1.95)	0.79 (0.37; 1.68)	0.540
	≥ 65	23	47	2.11 (1.05; 4.26)^*^	1.11 (0.44; 2.79)	0.828
BMI	Underweight	50	65	1	1	
	Normal weight	122	129	0.81 (0.52; 1.27)	0.97 (0.55; 1.70)	0.906
	Overweight	13	27	1.6 (0.75; 3.41)	1.07 (0.40; 2.85)	0.890
Alcohol	No	158	203	1	1	
	Yes	27	18	0.52 (0.28; 0.98)^*^	0.76 (0.34; 1.73)	0.519
Type of drug	Alkylating agent with anthracycline	68	28	1	1	
	Taxane regimen alone	22	91	10.05 (5.29; 19.07)^**^	2.99 (1.36; 6.55)^**^	**0.006**
	Taxane with combination	15	42	6.80 (3.26; 14.19)^**^	2.96 (1.25; 7.01)^*^	**0.014**
	Platinum alone and with a combination	42	38	2.20 (1.18; 4.09)^*^	1.29 (0.63; 2.63)	0.487
	Vinca alkaloids	25	17	1.65 (0.78; 3.52)	1.66 (0.68; 403)	0.266
	Others	13	5	0.93 (0.30; 2.87)	1.05 (0.31; 3.53)	0.939
Cycle of therapy	Cycle 2	78	40	1	1	
	Cycle 3	64	35	1.07 (0.61; 1.87)	1.11 (0.60; 2.11)	0.745
	Cycle 4	19	28	2.87 (1.43; 5.77)^**^	1.97 (0.88; 4.44)	0.101
	Cycle 5	14	57	7.94 (3.95; 15.95)^**^	2.65 (1.18; 6.00)^*^	**0.019**
	Cycle 6 and above	10	61	11.90 (5.51; 25.68)^**^	4.00 (1.67; 9.65)^**^	**0.002**

^*^ *p*-value < 0.05; ^**^ *p*-value < 0.01. CI, confidence interval; COR, crude odds ratio; AOR, adjusted odds ratio.

Bold values indicates p value of <0.05.

## Discussion

This study was conducted to assess the magnitude of chemotherapy-induced peripheral neuropathy and its associated factors among adult cancer patients undergoing chemotherapy in northwest Ethiopia oncology centers. Accordingly, the overall magnitude in this study was 54.4% (CI: 49.6–59.3). This finding is consistent with the studies conducted in Colombia (49.9%) (29), Minnesota, USA (52.7%) (6), and China (53.4%) (30).

The findings of this study are lower than those of studies conducted in Romania (68.09%) (31) and Kenya (83.6%) (17). The exclusion of participants with diabetic mellitus, HIV, and neurological diseases may be a possible cause of this difference (11). The smallest sample sizes of these two studies (163 and 67, respectively) could also contribute to this disparity. Additionally, differences in the measuring tools used may account for the variation. It is important to use a combination of scales to maximize the “pick up” rates of these tools, incorporating both patient‐reported outcomes and objective CIPN indicators, such as the TNS clinical version (32). This approach is supported by studies conducted by Molassiotis et al. in 2019 and Alberti et al. in 2014 (30, 33). Another consideration, especially for the study conducted in Kenya, is that it focused only on a single chemotherapeutic drug (cisplatin), which may be extremely toxic to peripheral nerves. It may also be related to the lifestyle of the respondents. Additionally, the finding is lower than the meta-analysis conducted by Serenty et al. in 2014, which reported a CIPN occurrence of 68.1% (5). This meta-analysis differentiated the occurrence of CIPN into the first, third, and subsequent sixth months, which are directly related to the cycle of therapy—a known risk factor for the development of CIPN. In contrast, this study did not consider these specific time points, which may explain the discrepancy.

In this study, the magnitude of CIPN is higher than in the French study, where the frequency was 31.3% (34). This difference may be explained by the fact that this study was conducted on cancer patients receiving chemotherapy, while the study in France was carried out after the completion of chemotherapeutic treatment. According to numerous researchers, although CIPN may persist for a long period, its presence decreases once chemotherapeutic treatment is completed (11, 35).

Regarding factors associated with the magnitude of CIPN, the odds of developing chemotherapy-induced peripheral neuropathy were higher among cancer patients in stage IV (AOR = 4.36) compared to those in stage I. This finding is in line with a study conducted by Kamgar et al. in the USA in 2021 (36).

Similarly, the odds of developing CIPN were higher among cancer patients receiving chemotherapy with comorbidities (AOR = 2.74) compared to those without comorbidities. This outcome is consistent with the research done by Miaskowski et al. in the USA in 2017 and 2018 (12, 37). The possible explanation is that conditions such as diabetes, hypertension, and renal impairment are associated with pre-existing damage in peripheral nerves. When chemotherapy is administered, these already-compromised nerves become more susceptible to further injury, exacerbating the severity of neuropathy. Additionally, comorbid conditions affecting kidney and liver function can impair the metabolism and clearance of chemotherapeutic drugs, leading to higher concentrations of neurotoxic agents in the bloodstream. This prolonged exposure further increases the risk and severity of CIPN. Furthermore, many chronic diseases are linked to elevated levels of inflammation and oxidative stress. When combined with chemotherapy, this cumulative burden significantly heightens the risk of nerve damage (38).

Regarding the therapeutic regimen, the odds of developing CIPN among cancer patients who received a taxane regimen were about three times more likely than those who received alkylating agent combined with anthracycline, specifically a combination of cyclophosphamide and doxorubicine. This finding is consistent with the studies conducted in Colombia and Romania (29, 31). This may be due to the fact that taxane drugs excessively disrupt normal axonal transport, leading to axonal swelling and subsequent peripheral nerve damage. Taxanes also accumulate in sensory neurons, particularly in the mitochondria, where they impair mitochondrial deoxyribonucleic acid (DNA) functions, reduce ATP production, and elevate oxidative stress. These combined effects lead to neuronal injury and degeneration, significantly contributing to the increase in CIPN. Taxanes increase reactive oxygen species (ROS) and trigger inflammation in peripheral nerves, overwhelming antioxidant defenses and damaging cellular components, thereby exacerbating neuropathy symptoms. They also interfere with neurotrophin pathways, particularly nerve growth factor (NGF), which reduces essential nerve support and hinders recovery from injury. Additionally, taxanes directly damage Schwann cells and myelin, impairing nerve conduction and leading to both sensory and motor neuropathy (10). This implies that cancer patients on taxane regimens require special attention and close follow-up.

Furthermore, patients who received taxane-based chemotherapeutic drugs in combination with a platinum-based regimen or other drug categories were 2.96 times more likely to develop CIPN compared to those who received alkylating agent with anthracycline. CIPN developed in 88.9% of patients receiving a carboplatin and docetaxel combination, which is an essential treatment for platinum-resistant ovarian cancer. It developed in 87.5% of patients receiving cisplatin with paclitaxel, commonly used for ovarian cancer, advanced non-small cell lung cancer (NSCLC), head and neck cancer, and esophageal carcinomas. In 80% of patients receiving carboplatin with paclitaxel, a combination used to treat multiple solid tumors, including ovarian, lung, breast cancer, and cervical cancers, CIPN developed, with minimal regard for scheduling to maximize response. It was seen in 70% of patients receiving cisplatin with docetaxel, a regimen used for certain types of head, neck, and stomach cancers. Lastly, 50% of patients receiving docetaxel with gemcitabine, a regimen used for treating leiomyosarcoma, other soft tissue sarcomas, and carcinomas of unknown origin, developed CIPN, with manageable toxicity (39). This shows that the magnitude of CIPN increases when chemotherapeutic drugs are used in combination (40). This may be due to the fact that, in addition to arresting the progression of cancer by inhibiting DNA synthesis through DNA cross-linkage or interfering with microtubules to block mitosis, leading to cancer cell apoptosis, these drugs also affect normal cells, particularly those of fast-growing tissues. As a result, numerous changes occur in the structure and function of neuronal and glial cells, leading to the activation and recruitment of immune cells. This process results in the release and elevation of proinflammatory cytokines (such as interleukins and chemokines), which cause nociceptor sensitization and hyperexcitability of peripheral neurons. These processes contribute to the development of neuroinflammation (10).

The number of chemotherapy cycles received was a strong risk factor in both in univariable and multivariable analyses in this study. Patients on the sixth cycle of chemotherapy were four times more likely to develop CIPN compared to those on the second cycle. The odds of developing CIPN among respondents on the fifth cycle of chemotherapy (AOR = 2.65) were higher compared to those on the second cycle. This implies that as the number of chemotherapy cycles increases, the risk of developing CIPN also increases, indicating that CIPN is time-dependent (5). The finding is consistent with the study conducted by Mazilu et al. in 2018 in Romania, which states that patients receiving more than four cycles of neurotoxic chemotherapeutic drugs have a higher chance of developing CIPN (31). This may be related to the fact that as the number of therapy cycles increases, the cumulative dose effect of neurotoxic agents also rises, thereby increasing the risk of nerve damage with each cycle. As the body accumulates these agents, the risk and severity of CIPN often escalate with cumulative doses rather than with single-cycle doses. This is primarily due to the limited recovery capacity of nerve cells between cycles. Repeated chemotherapy cycles progressively disrupt the structures of nerve cells, including microtubules, mitochondria, and Schwann cells, leading to axonal degeneration and impaired nerve function, which can manifest as sensory or motor neuropathy. Short intervals between cycles may not provide sufficient time for partial nerve repair, increasing the likelihood of sustained damage, while extended recovery periods may result in less severe CIPN. Furthermore, each chemotherapy cycle introduces additional oxidative stress and inflammatory responses in peripheral nerves, compounding cellular damage and overwhelming antioxidant defenses, which exacerbates neuropathy symptoms with each successive cycle (41).

Although it was not significant in this study, regular physical exercise has numerous benefits, including preventing general health problems and enhancing overall well-being. It can also help in the recovery from chemotherapy-induced peripheral neuropathy, particularly for those who experience significant distress from the condition, by improving blood circulation and strengthening nerve tissues through increased oxygen flow (42). A 2017 study in New York City showed that patients who spent more than 5 h/week of moderate-to-vigorous physical activity (MVPA) were 60% less likely to experience increased CIPN (43). In Ethiopia, the lack of significance of physical activity in studies on CIPN may be attributed to the population’s inherently active lifestyle. Many Ethiopians, including those in urban areas, regularly perform physical tasks, often through agricultural work or daily activities essential to meeting basic needs, typically without mechanized assistance. This incidental, widespread physical activity may reduce variability in physical activity levels, making it challenging to detect a distinct impact of physical activity on the risk or severity of CIPN across different groups.

## Limitation

Social desirability bias may have influenced responses, particularly regarding behavioral characteristics, despite the specific training provided to data collectors. Methodologically, future studies could benefit from using mixed methods, such as triangulating quantitative findings with qualitative data and employing prospective study designs, to more comprehensively identify risk factors for chemotherapy-associated peripheral neuropathy.

## Conclusion

This study is the largest study to date examining the magnitude of CIPN among cancer patients undergoing chemotherapy in the study area, providing valuable baseline data. Approximately half of the study participants experienced CIPN, highlighting that chemotherapy-induced peripheral neuropathy is a common adverse event among Ethiopian cancer patients treated with taxane-based regimen, platinum-based regimen, vinca alkaloids, and other widely used chemotherapeutic drugs, either alone or in combination. The type of drug regimen, therapy cycle, comorbidity, and cancer stage at diagnosis were identified as risk factors in this study. As we expand our understanding of the prevalence and risk factors of chemotherapy-induced peripheral neuropathy, certain targeted medications—whether preventive or treatment-related—may become more suitable based on the specific chemotherapeutic agents to which a patient is exposed. In general, establishing an effective CIPN monitoring and reporting system and raising awareness among healthcare professionals about the importance of CIPN reporting may aid in reducing and preventing the problem.

## Recommendation

The healthcare facilities with oncology center should establish more successful diagnostic methods and incorporate validated scales, such as EORTC assessment tools, either alone or in combination with other clinical tools, in the routine evaluation of all patients receiving chemotherapy in our environment. In addition, healthcare providers should focus not only on the patient’s medical issues during visits but also consider the patient’s overall health. To lessen the burden of CIPN on patient’s life, researchers should concentrate on improving our understanding of the mechanisms underlying the development of CIPN, as well as on preventive and treatment measures using both pharmacological and nonpharmacological therapies.

## Data Availability

The original data supporting the conclusions of this study are included in the article. Further inquiries can be addressed to the corresponding author and will be accommodated upon reasonable request, without undue restrictions.
